# Expected years lived with intimate partner violence: a new approach for public health

**DOI:** 10.1080/16549716.2021.1976442

**Published:** 2021-09-20

**Authors:** Amalia Gomez-Casillas, Mariona Lozano, Elisenda Rentería

**Affiliations:** Centre d’Estudis Demogràfics (CED-CERCA), UAB, Cerdanyola Del Vallès, Spain

**Keywords:** Intimate partner violence (IPV), years expected to live with IPV, violence against women, women’s health, international comparison

## Abstract

**Background:**

Intimate Partner Violence against women (IPV) is a major public health problem. However, mainstream indicators used in public health are not designed to fully capture the pervasive and enduring impact of IPV.

**Objective:**

We propose a new indicator that considers the burden of IPV in women during their middle life years, estimating the number of years that women are expected to live under IPV, and provide estimates for 151 countries.

**Methods:**

Prevalence rates of physical and sexual IPV for a given year are taken from the Global Database on the Prevalence of Violence Against Women. Annual period life tables are constructed using data from the World Population Prospects. We use Sullivan’s method to estimate partial life expectancy between the ages of 15 and 49 lived suffering from physical and sexual IPV in each country. The final indicator measures the number of years 15 to 49-year-old women are expected to live with IPV (YLIPV) in a given year.

**Results:**

Based on data from surveys representative of 92.0% of the global female population aged between 15 to 49, we find that ever-partnered women aged between 15 to 49 are expected to live 4.1 years (Low Bound: 2.3; Upper Bound: 7.1) suffering from violence during this age range. By regions, women are expected to suffer from IPV during 6.0 years (3.7–9.2) in Africa; 4.3 years (2.4–7.8) in Asia; 3.4 years (2.1–5.6) in Oceania; 2.6 years (1.5–4.2) in the Americas; and 1.7 years (0.9–3.1) in Europe.

**Conclusions:**

YLIPV is a useful indicator to display the burden of IPV. Similarly to the mainstream public health indicators rationale, YLIPV accounts for the time women are exposed to IPV during their lifespan and it is standardized by age exposure.

## Background

Intimate Partner Violence (IPV) is among the leading causes of morbidity and mortality in women with over a third of female homicides perpetrated by intimate partner [1]. Past studies have found that a third of ever-partnered women had suffered from physical and sexual IPV during their lives [2,3]. The global prevalence of IPV and its repetitive nature are related to victim’s co-residence or the close relationship with the perpetrator [4,5] and explain the long-lasting health consequences for the victim. This urges public health scholars to account for the global burden of IPV. Previous efforts to provide an overview of IPV’s implications on public health mainly involved accounting for IPV’s geographical extent [6], its effects on mortality – either due to homicide [1] or victims’ suicide attempts [7], health consequences [8], and its determinants [9].

Women suffering from IPV face multiple health problems. There is a wide range of negative consequences such as gastrointestinal, gynecological, and mental health problems [8,10]. Past studies have also shown a strong association between HIV infection and IPV [11,12]. Among the psychological consequences, IPV is associated with depression, post-traumatic stress disorder, anxiety, memory loss, and sleep disorders among others [13].

Since the World Health Organization (WHO) declared IPV as a key public health issue [14], it has gained increasing visibility in public health studies, and it has recently been incorporated in the Global Burden of Disease (GBD) estimates of Disability-Adjusted Life-Years (DALY) and Health-Adjusted Life Expectancy (HALE) [15,16]. Despite these remarkable efforts to explain the burden of IPV, neither DALY nor HALE indicators can fully account for the pervasive and enduring IPV experience and its impact on women’s quality of life, as they particularly measure the disabling effects of IPV. The burden of IPV is neither limited to a sequence of assaults [17], nor fully explained by its health consequences alone. Additionally, IPV also wrecks women’s lives through a day by day eroding experience of increasing fear, constant threats which lead victims to be continuously alert and to feel despair, guilt, shame, and confusion [17–19]. IPV is a repetitive cycle that begins with a first phase of tension building, leading to a second phase of violent episodes, and a final phase of loving contrition [20]. In this repetitive cycle, violence tends to escalate, turning women’s fear into terror, and generating acute feelings of disempowerment and loss of self-respect. This process constitutes an ‘emotional career’ [18] where IPV erodes women’s quality of life daily.

The objective of this paper is to propose an easy-to-read indicator that fully captures the burden of IPV in a woman’s life. It measures the number of years that women are expected to live suffering from IPV. While life expectancy in most societies has increased, there is a debate whether the current indicators capture the quality of these years gained beyond just the number [21]. This process has led to the development of indicators such as Healthy Life Expectancy (HLE), which measures the number of years that a person is expected to live without activity limitation or health problems. To illustrate the gradual incorporation of quality indicators in the policymaking agenda, it is worth mentioning the incorporation of HLE to monitor progress in European policies, such as the 2000 Lisbon strategy [22]. This paper builds on these initiatives by proposing a new indicator called Years expected to Live with Intimate Partner Violence (YLIPV), that aims to highlight the persistence of IPV in women’s lifespan, and complements previous public health indicators that did not fully capture the pervasive condition of IPV. YLIPV could be especially helpful for policy design and funding allocation, as well as for increasing awareness of all the possible unmeasured burdens of IPV in women’s quality of life. We use YLIPV to show international inequalities in 151 countries based on the modelled prevalence data recently released by WHO.

## Methods

### Data sources

Data for prevalence of physical and sexual intimate partner violence are taken from the WHO’s Global Database on the Prevalence of Violence Against Women [23]. These data provide aggregated estimates of the proportions of ever-partnered 15 to 49-year-old women and girls by five-year age groups. These women and girls have been subjected to physical and/or sexual violence by a current or former intimate partner in the previous 12 months. The Inter Agency Working Group on Violence against Women Estimation and Data supported by the Technical Advisory Group (TAG) developed a statistical modeling framework [24] aiming to provide prevalence estimates at global, regional, and national levels gathered in the WHO’s Global Database. Based on a systematic review of studies conducted between 2000 and 2018 (available by 2019), the WHO and the associated groups created population-based nationally and sub-nationally representative database. Further, the prevalence of IPV is assessed using acts-based IPV measures (act-based questions entail asking women if they suffer violent acts such as pushing, grabbing, kicking or other violent behaviours). In this regard, the collected data on IPV were adjusted using complex models to finally obtain a unique estimate for each of the relevant measures that was representative for the whole period. More information about the inclusion criteria for the studies and the procedure underlying the modeled prevalence is available in the WHO related documents [24,25]. Available data refer to ever-partnered women and girls aged from 15 to 49 years old. Perpetrators are current or former intimate partners involved in any formal or informal relationship (marriage, cohabitation or any other form of union).

Country-wise female life tables (organized in periods of five years) are taken from the World Population Prospects database [26]. We select the 2010–2015 life table for analysis as it matches with the median point of the period of reference of IPV prevalence data for all countries [25]. We estimate the regional aggregates for the Sustainable Development Goals Super Regions, which are Africa, the Americas, Asia, Europe, and Oceania. To estimate the expected number of years lived with IPV (YLIPV) for regional aggregates we need the proportion of ever-partnered women in each country. However, this information was not available for all the studied countries. Thus, we use data on female population from the World Population Prospects (July the 1^st^ 2012) [27] to weight each country and calculate the regional and global mean estimates of YLIPV. These weights are designed for female populations aged from 15 to 49 years old. Therefore, global results for YLIPV were based on the 92.0% of the female population worldwide in 2012. To double check that the regional means were not biased by weighted estimates, we also weight results using the female population currently married or cohabiting [28]. The relative difference between the two was 2.4%, and it goes up to 11.1% per continent (see the supplementary materials).

### Statistical analysis

We calculate the number of years the female population between 15 and 49 years old is expected to live under IPV using the Sullivan’s method [29]. We analyze data for 151 countries and territories and estimate the burden of IPV on women’s lives. Sullivan’s method uses prevalence data (IPV_x_) on ever-partnered women that have experienced physical and/or sexual violence in the last 12 months in each of the five-year age groups (x, x + 5), applied to the person-years indicator (${\;}_{5}^{\;}L_{x}$) to re-estimate the life expectancy spent under IPV or YLIPV [28]. Formally, our indicator is calculated as:

(1) $$YLIPV=\frac{1}{l_{x}}\overset{50}{\underset{x=15}{\sum }}IPV_{x}\cdot {\;}_{5}^{\;}L_{x}$$

where $l_{x}$ is the number of survivors at exact age x.

We estimate the partial life expectancy from ages 15 to 49 (more details can be found in the supplementary materials) because the prevalence data are available for this age range only. We also calculate the proportion of life expectancy between age 15 and 49 spent under IPV (life expectancy while living with violence). Lower and upper bound estimates of IPV for each country are obtained using the 95% uncertainty intervals (UI) that WHO provides along with the prevalence estimates. We opt for this approach to overcome the limitation of the WHO modelled samples. Lower bound and upper bound estimates are reported as LB and UB, respectively.

## Results

Based on prevalence data from 151 countries, women in our study are expected to live with IPV (YLIPV) for 4.1 years based on population weighted mean (LB 2.3 – UB 7.1). Table 1 shows that this estimate is obtained for 92.0% of the world total reference population of ever-partnered females aged between 15 and 49. YLIPV is 4.2 years if results are weighted using the total population of women who are either currently married or in a union. Hence, the difference between both weighting strategies is merely 0.1 years. We consider this difference to not bias our results. Our data represent 90.7% of women in the African continent, 68.9% of the women in the European population, 99.4% of the women in South and North America, 94.3% of the women in the Asian population, and 98.0% of the women in the population from Oceania.

**Table 1.** Regional prevalence of intimate partner violence and number of years expected to live with intimate partner violence (YLIPV) among ever–partnered females, 2000–2018

Region	Population coverage (women aged from 15 to 49 years old)	YLIPV (years)
Summary for 151 countries	92.0%	4.1 (2.3–7.1)
Africa (based on 43 countries)	90.7%	6.0 (3.7–9.2)
Americas (based on 28 countries)	99.4%	2.6 (1.5–4.2)
Asia (based on 32 countries)	94.3%	4.3 (2.4–7.8)
Europe (based on 38 countries)	68.9%	1.7 (0.9–3.1)
Oceania (based on 10 countries)	98.0%	3.4 (2.1–5.6)

Note: Low and Upper Bound estimates in brackets. Methods section provides more information on how these measures are estimated.

Source: authors’ elaboration.

In Africa, women are expected to suffer from IPV for 6 years (LB 3.7-UB 9.2) on average.

**Figure 1.**
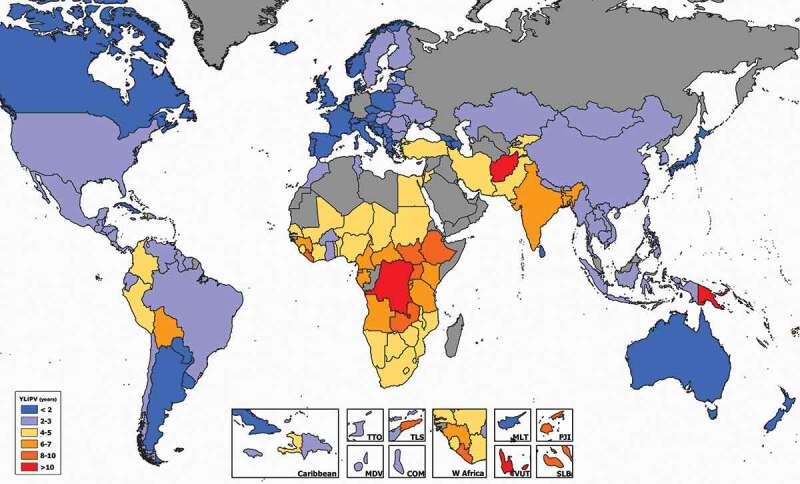
Expected years suffering from IPV estimated as number of Years expected to Live with Intimate Partner Violence (YLIPV) among ever-partnered females aged 15 to 49 years, for 151 countries and territories, 2000–2018

Notes: TTO: Trinidad and Tobago; MDV: Maldives; TLS: Timor-Leste; COM: Comoros; MLT: Malta; VUT: Vanuatu; FJI: Fiji; SLB: Solomon Islands.

Source: Authors’ elaboration.

Figure 1 indicates that women are most affected in the Democratic Republic of Congo, where they are expected to live 11.4 years (LB 7.4-UB 16.0) with IPV, which equals 34.9% (LB 22.6-UB 48.9) of their 15–49 lifespan (country-estimations are available in the supplementary material Table 2A). In Equatorial Guinea, Zambia, Ethiopia, Liberia, South Sudan, and Uganda YLIPV is over 8 years on average, and women in these countries are expected to live between 25.5% and 28.2% of their 15–49 lifespan as victims of physical and sexual IPV.

In South and North America women are expected to live 2.6 years (LB 1.5-UB 4.2) suffering from IPV. The highest figures of YLIPV are recorded in Bolivia, where women are expected to live 6.4 years (LB 3.9-UB 9.6) with IPV, which equals 18.8% (LB 11.5-UB 28.2) of their 15–49 life expectancy. Women in Colombia and Haiti are expected to live 4.1 years suffering from IPV (11.9% and 12.5% of the 15–49 lifespan, respectively) as shown in Figure 1. The lowest numbers are found in Canada (1.0 year; LB 0.6-UB 1.6).

In Asia, women are expected to suffer from IPV for 4.3 years (LB 2.4-UB 7.8) between the ages of 15–49. As shown in Figure 1, we find that the highest prevalence of IPV is estimated in Afghanistan, where women are expected to live 11.4 years under IPV between the ages of 15 and 49. This represents 34.3% (LB 21.6-UB 49.9) of their life expectancy between these years. In India women are expected to live 6.2 (LB 3.8-UB 9.6) years suffering from IPV, which equals 18.3% (LB 11.2-UB 28.3) of their 15–49 life expectancy. In Bangladesh, YLIPV is 7.9 (LB 5.1-UB 11.6), and 9.4 (LB 6.3-UB 13.5) in Timor-Leste. In Singapore, Japan, and Sri Lanka, YLIPV is recorded under 2.0. In China, women lived 2.8 years (LB 1.1-UB 6.6) on average as victims of this form of violence, which equals 8.1% of their 15–49 lifespan (LB 3.2-UB 19.0).

In Europe, women suffer from violence for 1.7 years (LB 0.9-UB 3.1) on average. As shown in Figure 1, the highest numbers of YLIPV are found in the Republic of Moldova (3 years; LB 1.7-UB 5.2), Ukraine (2.9; LB 1.7-UB 4.6), and Finland (2.9; LB 2.0-UB 4.0). These equal, respectively, to 8.7%, 8.4%, and 8.3% of women’s 15–49 lifespan. The lowest numbers are found in Switzerland (0.7 years; LB 0.3-UB 1.4), and Iceland (0.9; LB 0.6-UB 1.6).

In Oceania, women are expected to live 3.4 years (LB 2.1-UB 5.6) of their lives suffering from IPV. In Australia, women are expected to live 1.0 year (LB 0.6-UB 1.7) as victims of physical and sexual IPV, but this goes up to 10.3 (LB 6.3-UB 15.4) and 10.1 years (LB 5.4-UB 16.7) in Papua New Guinea and Vanuatu, respectively.

## Discussion

Our results indicate that, on average, women are expected to suffer from IPV for 4.1 years, but there are important differences in the experience of IPV amongst the 151 countries. Global inequalities in YLIPV found in this study point to a difference of 10.7 years of life between the highest and the lowest exposure to this type of violence. The longest time exposure to IPV is found in Afghanistan and in the Democratic Republic of Congo (YLIPV in both countries is 11.4 years), while the lowest is found in Switzerland (0.7). Overall, our data confirm the established pattern of IPV exposure, with women in the Middle East and Southern Asia being exposed to a larger prevalence of IPV. This translates into a greater proportion of their lives spent suffering from this type of violence.

Previous studies on IPV have pointed out that the highest rates of physical and/or sexual IPV from the age of 15 were found in Central Sub–Saharan Africa [2,3]. Our study confirms that certain central African countries also have the highest prevalence rates of IPV and the largest YLIPV numbers. However, the comparison between our results and the previous studies comparing cross-country IPV rates is challenging due to data coverage, the type of indicator considered, and the complex relationships between lethal and non-lethal IPV victimization. In addition, the relationship between life expectancy and IPV prevalence for a given year is not straightforward because it depends on union formation and dissolution dynamics [5], the probability of women escaping from the violent relationship [5], and the probability of them being murdered by their partners. In those countries where IPV homicide [1] rates are higher, prevalence may be expected to be downsized because murdered women are not captured in the samples. IPV can also cause the victim’s death due to suicide [7], which also reduces IPV prevalence. Mortality rates among males may also affect these results. One could expect lower rates of IPV for populations with higher proportions of female widows, and larger deaths among perpetrators. In addition, YLIPV can be lower in those countries with higher mortality rates in general, because life expectancy in those countries will be shorter. Nonetheless, using the percentage of YLIPV over Life Expectancy amends this problem. Finally, we use all women between 15 and 49 as our reference population, and this might alter the results as well. Women who were in a union at the time of the survey or who have recently separated are at a higher risk than those who had experienced a union dissolution a long time ago.

Cross-country comparisons of YLIPV show that women in countries with lower socioeconomic conditions have spent a larger number of years suffering from IPV. This is contrary to the results found by the Global Burden of Disease studies [15,16] that estimated HALE at a global scale while considering all diseases. Although their estimates cannot be compared to ours because they use different indicators and weighting structure, the GBD study shows that years of functional health lost, estimated by subtracting HALE from Life Expectancy, are higher in high-income countries because these countries combine higher life expectancies and a greater prevalence of less fatal diseases. Hence, women live longer but also more years in poorer health. With IPV estimates, the association goes in the opposite direction, and the countries with lower socioeconomic conditions are showing a larger number of years suffering IPV, calling attention to the fact that women’s health in low-income countries is being deteriorated by this type of violence.

This paper advances the current knowledge on IPV by suggesting a synthetic indicator, YLIPV, that shows in a single number the impact of this form of violence in each of the studied countries, contributing to the literature on measuring gender–based violence in two ways. First, the relevance of our indicator lies in its capacity to display the burden of IPV, similarly to the mainstream public health indicators rationale. Our indicator accounts for the time women are exposed to IPV during their lifespan and is standardized by age exposure. Hence, YLIPV captures the magnitude and relevance of IPV as a comprehensive and persistent experience contributing to the global description of IPV. Second, while several studies have already addressed IPV as a global burden from different perspectives, our contribution lies in measuring, for the first time, the length of this type of violence during the central years of life. This allows us to better capture the impact of IPV in women’s lives. As a result, YLIPV complements other mainstream indicators improving public health policy assessment.

This study, however, comes with some limitations, mostly related to cross-country comparability of IPV prevalence. Differences in survey design methodology, definitions, questionnaires, question wording and survey designs hamper the comparability of the results [4,24]. These restraints led the WHO to develop a methodological framework for providing modelled estimates based on a systematic review of prevalence studies. These studies need to meet various minimum requirements, such as being representative at a national or subnational level and using acts-based measures, among others [24;25]. Despite these methodological improvements [24], self-disclosure related problems remain a drawback for international comparisons. IPV is widely recognized to be underreported due to its stigma, shame, fear, and recall biases [30]. Underestimation of the real prevalence is also linked to surveys using small sample sizes, skewed sample frames, the method used to contact the respondents, questions wording, the number of questions in the questionnaires and the order in which they are asked [4,30], sex of the interviewers, their skills, attitudes and their training to address these sensitive issues [31]. Moreover, in the prosecution of IPV cases, cultural acceptance and law enforcement play a role in IPV disclosure. Thus, prevalence results must be understood as low bound estimations of physical and sexual IPV. Also, in order to display improved estimates, women aged 50 or over should be incorporated in the analysis. For future analysis, while this indicator considers a great deal of the gender-based violence victimization, other forms of violence should be further incorporated. Finally, relying in the lower and upper bounds of each age group could be artificially widening the uncertainty range of our estimates, meaning that the real uncertainty levels could be, in fact, lower. At the same time, we did not consider any uncertainty coming from the life table estimates.

To sum up, this study suggests a new indicator to measure global trends in IPV. YLIPV accounts for the number of years that women are expected to live with IPV during their lives, and it has the advantage of standardizing country-level data to display global inequalities, which makes comparison between countries and over time easier. In other mainstream indicators, such as HALE, IPV is characterized only by its health consequences, underestimating the burden of the ‘emotional career’ [18] of IPV over women’s lives. In conclusion, YLIPV contributes to raise awareness of IPV’s global scope and constitutes a first step to integrate the comprehensive and pervasive experience of IPV.

## Supplementary Material

Supplemental Material

## Paper context

This paper introduces a new synthetic indicator, Years expected to Live with Intimate Partner Violence (YLIPV), which aims to capture the pervasive and enduring burden of Intimate Partner Violence (IPV). It indicates the number of years women are expected to live with IPV and is intended to be useful for public health policy, and to raise awareness of the IPV exposure. Additionally, we estimate YLIPV for women aged 15 to 49 years in 151 countries.
