# Supplementary material for: Expected years lived with intimate partner violence: a new approach for public health
Source: Glob Health Action. 2021 Sep 20;14(1):1976442. doi: 10.1080/16549716.2021.1976442 (PMC8462847; doi:10.1080/16549716.2021.1976442)
Supplement: Supplemental Material [file ZGHA_A_1976442_SM6681.pdf]

Supplementary materials

Supplement to: Gómez-Casillas A, Lozano M, Rentería, E. Expected years lived with intimate partner violence: a new approach for public health. Global Health Action 2021
https://doi.org/10.1080/16549716.2021.1976442

1. Estimation of the partial life expectancy between age 15 and age 49.

To estimate the percentage of IPV life expectancy between the ages of 15-49, we need to calculate in a first step the partial 15-49 life expectancy. This is calculated as follows:

$$e_{15}^{50} = e_{50} - e_{15} = \frac{1}{l_{15}} \sum_{15}^{w} {}_nL_{50} - \frac{1}{l_{50}} \sum_{15}^{w} {}_nL_{15} \quad (1A)$$

where e_x is the life expectancy from age x until the end of life (w age group), which is calculated by dividing all the person-years (${}_nL_x$) from that age x and beyond by l_x, which is the number of survivors at exact age x.

Then, we divide the YLIPV (formula in the text) by the life expectancy between age 15 and 49 (e_{15}^{50}), to obtain the proportion of life expectancy expected to be lived with IPV if all women from age 15 would experience the same prevalence of IPV that are observed in that given year.

2. Weighting structure

The paper displays population weighted global aggregates. For this purpose, we use the total female population aged from 15 to 49 for 2012 [1]. Although the accurate weighting structure would consider the number of ever-partnered women, these results are not available. We, therefore, conduct a robustness check estimating the regional and world aggregates weighted using the share of population currently married or in union for 2012 [2]. Table 1A indicates the variation in the results per regions and worldwide.

Table 1A. Number of Expected Years lived suffering Intimate Partner Violence (YLIPV) weighted following total female population and female population married or in union aged between 15 and 49.

World region	Weighting structure based on total female population (aged from 15–49)	Weighting structure based on female population married or in union (aged from 15–49)	Relative difference
World	4.1	4.2	2.4%
Africa	6.0	6.0	0.0%
Asia	4.3	4.4	2.3%
Americas	2.6	2.6	0.0%
Oceania	3.4	3.8	11.1%
Europe	1.7	1.7	0.0%

3. Data information and estimates by country

Table 2A. Results on the Years Lived with Intimate Partner Violence (YLIPV) for 151 countries

		Data on IPV prevalence	Life table estimates	
**Sustainable Development Goals Super Regions**	**Country***	**Source used by the World Health Organization (WHO)[3] to provide the modelled estimates**	**Years expected to Live with IPV (YLIPV) (ages 15–49) (lower and higher bound estimates)**	**Life expectancy 15–49 suffering IPV (%) (lower and higher bound estimates)**
Africa	Angola	Demographic and Health Survey [DHS] (2017)	7.8 (4.6-12.5)	23.7% (13.9-37.9)
Africa	Benin	Demographic and Health Survey [DHS] (2019)	4.7 (2.6-8.0)	14.3% (7.9-24.3)
Africa	Botswana	Gender based violence indicator study (2012); Risk factors for domestic physical violence: national cross-sectional household surveys in eight southern African countries (Andersson et al.) (2007)	5.5 (3.0-9.5)	16.6% (9.1-28.8)
Africa	Burkina Faso	Enquête Démographique Et De Santé Et À Indicateurs Multiples (EDSBF-MICS IV) (2012)	3.6 (1.9-6.5)	11.1% (5.9-20.0)
Africa	Burundi	Demographic and Health Survey [DHS] (2017)	7.0 (4.1-11.2)	21.7% (12.7-34.7)
Africa	Cabo Verde	Troisième Enquête Démographique et de Santé reproductive (IDSR III) (NA); Inquérito Demográfico E De Saúde Reprodutiva (DHS) (2008)	3.7 (2.2-6.3)	10.7% (6.4-18.2)
Africa	Cameroon	Enquête par grappes à indicateurs multiples (MICS5) 2014 (2015); Demographic and Health Survey [DHS] (2012); Demographic and Health Survey [DHS] (2005)	6.8 (4.4-10.0)	21.2% (13.7-31.1)
Africa	Central African Republic	Multiple Indicator Cluster Survey (MICS)-3 (2009)	6.1 (3.3-10.2)	20.0% (10.8-33.5)
Africa	Chad	Demographic and Health Survey [DHS] (2016)	4.9 (2.8-8.3)	15.7% (9-26.6)
Africa	Comoros	Demographic and Health Survey [DHS] (2014)	2.8 (1.3-5.5)	8.4% (3.9-16.5)
Africa	Côte d'Ivoire	Demographic and Health Survey [DHS] (2013)	5.0 (2.8-8.7)	15.9% (8.9-27.6)
Africa	Democratic Republic of the Congo	Demographic and Health Survey [DHS] (2014); Association of Sexual Violence and Human Rights Violations With Physical and Mental Health in Territories of the Eastern DRC (2010); Demographic and Health Survey [DHS] (2008)	11.4 (7.4-16.0)	34.9% (22.6-48.9)
Africa	Egypt	Egypt Economic Costs of GBV Survey (2015); Demographic and Health Survey [DHS] (2015); Sociodemographic associations of intimate partner violence against women in a rural area, El-Minia governorate, Egypt, 2010 (2012); Prevalence and Effects of Violence Against Women in a Rural Community in Minia Governorate, Egypt (2011); Demographic and Health Survey [DHS] (2006); World Studies of Abuse in the Family Environment (WorldSAFE) (2004)	5.1 (3.3-7.7)	14.8% (9.6-22.3)
Africa	Equatorial Guinea	Demographic and Health Survey [DHS] (2012)	9.1 (5.1-14.6)	28.2% (15.8-45.3)
Africa	Eswatini	Risk factors for domestic physical violence: national cross-sectional household surveys in eight southern African countries (Andersson et al.) (2007)	5.2 (2.8-8.9)	17.6% (9.5-30.1)

Africa	Ethiopia	Disparities in Intimate Partner Violence among Currently Married Women from Food Secure and Insecure Urban Households in South Ethiopia: A Community Based Comparative Cross-Sectional Study (Andarge, E. & Shiferaw, Y.) (2018); Demographic and Health Survey [DHS] (2017); Domestic violence and its predictors among married women in reproductive age in Fagitalekoma Woreda, Awi zone, Amhara regional state, North Western Ethiopia (Semahegn, A., Belachew, T., Abdulahi, M.) (2013); Magnitude and Correlates of Intimate Partner Violence against Women and Its Outcome in Southwest Ethiopia (Deribe et al.) (2012); WHO Multi-Country Study (WHO MCS) (2005)	8.5 (5.4-12.6)	25.6% (16.3-38.0)
Africa	Gabon	Demographic and Health Survey [DHS] (2013)	7.1 (4.0-12.1)	21.5% (12.1-36.6)
Africa	Gambia	Demographic and Health Survey [DHS] (2014)	3.1 (1.6-5.8)	9.5% (4.9-17.8)
Africa	Ghana	Poor mental health in Ghana: who is at risk? (Sipsma et al.) (2013); Demographic and Health Survey [DHS] (2009)	3.3 (1.8-5.7)	10.1% (5.5-17.4)
Africa	Guinea	L'enquête nationale sur les Violences Basées sur le Genre (VBG), Guinée, 2016 (NA)	6.6 (3.6-10.8)	20.4% (11.2-33.5)
Africa	Kenya	Demographic and Health Survey [DHS] (2015); Demographic and Health Survey [DHS] (2010); Demographic and Health Survey [DHS] (2004)	7.4 (4.9-10.7)	22.4% (14.8-32.4)
Africa	Lesotho	Risk factors for domestic physical violence: national cross-sectional household surveys in eight southern African countries (Andersson et al.) (2007); Sexual Violence in Lesotho (Brown, L., Thurman, T., Bloem, J. & Kendall, C.) (2006)	4.8 (2.4-9.0)	16.4% (8.2-30.7)
Africa	Liberia	Association of exposure to intimate-partner physical violence and potentially traumatic war-related events with mental health in Liberia (Vinck, P., & Pham, P.P.) (2013); Association of Combatant Status and Sexual Violence With Health and Mental Health Outcomes in Postconflict Liberia (2008); Demographic and Health Survey [DHS] (2008)	8.5 (5.2-12.9)	25.8% (15.8-39.1)
Africa	Malawi	Demographic and Health Survey [DHS] (2017); Demographic and Health Survey [DHS] (2011); Risk factors for domestic physical violence: national cross-sectional household surveys in eight southern African countries (Andersson et al.) (2007); Demographic and Health Survey [DHS] (2005)	5.3 (3.5-7.7)	16.2% (10.7-23.5)
Africa	Mali	Demographic and Health Survey [DHS] (2019); Demographic and Health Survey [DHS] (2013); Demographic and Health Survey [DHS] (2006)	5.7 (3.9-8.4)	17.7% (12.1-26.0)
Africa	Morocco	Enquête nationale sur la prévalence de la violence à l'égard des femmes au Maroc (ENPVF) (2012)	3.6 (2.0-6.4)	10.4% (5.8-18.5)
Africa	Mozambique	Demographic and Health Survey [DHS] (2018); Demographic and Health Survey [DHS] (2013); International Violence Against Women Survey (IVAWS) (2008); Risk factors for domestic physical violence: national cross-sectional household surveys in eight southern African countries (Andersson et al.) (2007)	5.1 (3.3-7.4)	16.3% (10.5-23.6)
Africa	Namibia	Demographic and Health Survey [DHS] (2014); Risk factors for domestic physical violence: national cross-sectional household surveys in eight southern African countries (Andersson et al.) (2007); WHO Multi-Country Study (WHO MCS) (2005)	5.1 (3.1-8.3)	15.8% (9.6-25.7)
Africa	Niger	Violences Basées sur le Genre au Niger (2015)	4.2 (2.4-7.0)	12.8% (7.3-21.4)

Africa	Nigeria	Demographic and Health Survey [DHS] (2019); Prevalence and Factors Associated with Intimate Partner Violence among Married Women in an Urban Community in Lagos State, Nigeria (Onigbogi, M.O., Odeyemi, K.A., Onigbogi, O.O.) (2015); Intimate Partner Violence among Women of Child Bearing Age in Alimosho LGA of Lagos State, Nigeria (Adegbite, OB & Ajuwon, AJ) (2015); Demographic and Health Survey [DHS] (2014); Intimate partner violence in southwestern Nigeria: are there rural-urban differences? (Balgoun, M., Owoaje, E.T., Fawole, O.I.) (2012); Demographic and Health Survey [DHS] (2009)	4.0 (2.6-5.9)	12.9% (8.4-19.0)
Africa	Rwanda	Demographic and Health Survey [DHS] (2016); Women are considerably more exposed tointimate partner violence than men in Rwanda:results from a population-based, cross-sectionalstudy (Umubyeyi, a., Mogren, I., Ntaganira, J. & Krantz, G.) (2014); Demographic and Health Survey [DHS] (2012); Demographic and Health Survey [DHS] (2006)	7.7 (5.0-11.1)	23.0% (14.9-33.1)
Africa	Sao Tome and Principe	Demographic and Health Survey [DHS] (2010)	6.0 (3.2-10.5)	17.6% (9.4-30.8)
Africa	Senegal	Demographic and Health Survey [DHS] (2018)	4.1 (2.2-7.1)	12.2% (6.5-21.1)
Africa	Sierra Leone	Demographic and Health Survey [DHS] (2014)	5.9 (3.3-9.7)	19.1% (10.7-31.5)
Africa	South Africa	Demographic and Health Survey [DHS] (2019); Gender Based Violence Indicators Study (2013)	4.3 (2.3-7.4)	13.3% (7.1-22.8)
Africa	South Sudan	Violence against conflict affected women and girls in South Sudan (Ellsberg et al.) (2018)	8.4 (3.8-15.2)	26.5% (12.0-47.9)
Africa	Sudan	Domestic violence against women in Eastern Sudan (Ali, A.A., Yassin, K., Omer, R.) (2014)	5.4 (2.7-10.0)	16.2% (8.1-30.0)
Africa	Togo	Demographic and Health Survey [DHS] (2014)	4.0 (2.1-7.0)	12.4% (6.5-21.6)
Africa	Tunisia	Enquête Nationale Sur La Violence À L'égard Des Femmes En Tunisie (2011)	3.6 (1.7-6.9)	10.4% (4.9-19.9)
Africa	Uganda	Demographic and Health Survey [DHS] (2018); Combined intimate partner violence and HIV/AIDS prevention in Rural Uganda: Design of the SHARE intervention strategy (Wagman et al.) (2016); Demographic and Health Survey [DHS] (2012); Demographic and Health Survey [DHS] (2007); Community HIV Epidemiological Research - 2 (CHER-2): Domestic violence in rural Uganda: evidence from a community-based study (Koenig et al. 2003) (2003)	8.3 (5.6-11.7)	25.5% (17.2-36.0)
Africa	United Republic of Tanzania	Demographic and Health Survey [DHS] (2016); Demographic and Health Survey [DHS] (2011); WHO Multi-Country Study (WHO MCS) (2005); Gender inequality and intimate partner violence among women in Moshi, Tanzania (McCloskey, L.A., Williams, C., Larsen, U.) (2005); Rape against women: The magnitude, perpetrators and patters of disclosure of events in Dar es Salaam, Tanzania (Muganyizi, P.S.) (2004)	7.8 (5.1-11.1)	23.7% (15.5-33.8)
Africa	Zambia	Demographic and Health Survey [DHS] (2015); Demographic and Health Survey [DHS] (2009); Risk factors for domestic physical violence: national cross-sectional household surveys in eight southern African countries (Andersson et al.) (2007)	8.7 (5.8-12.4)	26.9% (17.9-38.3)
Africa	Zimbabwe	Demographic and Health Survey [DHS] (2016); Demographic and Health Survey [DHS] (2012); Risk factors for domestic physical violence: national cross-sectional household surveys in eight southern African countries (Andersson et al.) (2007); Demographic and Health Survey [DHS] (2007)	5.7 (3.7-8.3)	18.1% (11.8-26.4)
Americas	Argentina	Estudio Nacional sobre Violencia contra las Mujeres (2019); Primer estudio nacional sobre violencias contra la mujer (2017)	1.6 (0.8-3.0)	4.6% (2.3-8.7)

Americas	Belize	Belize Public Health Survey (2015)	2.8 (1.1-6.2)	8.2% (3.2-18.1)
Americas	Bolivia (Plurinational State of)	Encuesta nacional de percepciones sobre situacion de violencia contra las mujeres, trata y tráfico en Bolivia (2015); Encuesta nacional de percepciones sobre situacion de violencia contra las mujeres, trata y tráfico en Bolivia (2015); Encuesta Nacional de Demografía y Salud (DHS) (2009); Encuesta Nacional de Demografía y Salud (DHS) (2004)	6.4 (3.9-9.6)	18.8% (11.5-28.2)
Americas	Brazil	Violência doméstica e familiar contra a mulher (2017); Gender-based violence and sexual and reproductive health among low-income youth in three Brazilian cities (Chacham, A.S., Simão, A.B., Caetano, A.J.) (2016); Prevalence and factors associated with intimate partner violence among women in Recife/Pernambuco, Brazil (Barros et al.) (2016); Brazilian Alcohol and Drug Survey (2016); Brazilian Alcohol and Drug Survey (2016); Prevalência de violência física por parceiro íntimo em homens e mulheres de Florianópolis, Santa Catarina, Brasil: estudo de base populacional (2015); Brazilian National Alcohol Survey (Zaleski) (2010); Sexual Behaviour And Perceptions Of AIDS, CEBRAP (2008); WHO Multi-Country Study (WHO MCS) (2005)	2.4 (1.4-3.7)	7.0% (4.1-10.7)
Americas	Canada	General Social Survey: Family violence in Canada (2016); General Social Survey: Family violence in Canada (2011); Psychological and health sequelae of child sexual, physcial and psychological abuse (Bouchard, E-M., Tourigny, J. J., Hébert, M. & Cyr, M.) (2008); General Social Survey: Family violence in Canada (2006); Statistics Canada (Juristat_Statistique Canada) (2004); A Survey on Violence Against Female Partners in Québec, Canada (Rinfret-Raynor, M., Riou, A., Cantin, S., Drouin, C., & Dubé, M.) (2004)	1.0 (0.6-1.6)	2.9% (1.7-4.6)
Americas	Chile	Tercera encuesta nacional de violencia intrafamiliar contra la mujer y delitos sexuales (2017); Encuesta nacional de victimizacion por violencia intrafamiliar y delitos sexuales (2013); Encuesta nacional de victimizacion por violencia intrafamiliar y delitos sexuales (2009); World Studies of Abuse in the Family Environment (WorldSAFE) (2004)	2.0 (1.1-3.7)	5.8% (3.2-10.7)
Americas	Colombia	Demographic and Health Survey [DHS] (2015); DHS-Encuesta nacional de demografía y salud ENDS2010 (2011); Demographic and Health Survey [DHS] (2005); Encuesta Nacional De Demografía Y Salud (ENDESA/DHS) (2000)	4.1 (2.1-7.5)	11.9% (6.1-21.7)
Americas	Costa Rica	International Violence Against Women Survey (IVAWS) (2008)	2.5 (1.2-4.9)	7.2% (3.5-14.1)
Americas	Cuba	Encuesta Nacional sobre Igualdad de Género ENIG-2016 (2018)	1.8 (0.8-3.5)	5.2% (2.3-10.1)
Americas	Dominican Republic	Encuesta demográfica y de salud [DHS] (2014); Encuesta demográfica y de salud [DHS] (2008); Encuesta demográfica y de salud [DHS] (2003)	3.3 (2.1-5.2)	9.6% (6.1-15.2)
Americas	Ecuador	Encuesta nacional sobre relaciones familiares y violencia de genero contra las mujeres (2014); Encuesta Demográfica y De Salud Materna e Infantil (ENDEMAIN) (2005)	2.9 (1.7-4.9)	8.4% (4.9-14.3)
Americas	El Salvador	Encuesta Nacional de violencia contra la mujer (2018); Violencia contra las mujeres en el Salvador (2015); Encuesta Nacional De Salud Familiar (FESAL)(DHS) (2009); Encuesta Nacional De Salud Familiar (FESAL) (DHS) (2003)	2.0 (1.2-3.0)	5.8% (3.5-8.7)
Americas	Grenada	Grenada Women’s Health And Life Experiences Study (2018)	2.9 (1.4-5.2)	8.5% (4.1-15.2)
Americas	Guatemala	Encuesta Nacional De Salud Materno Infantil (RHS) (MSPAS INE Segeplan) (2017); (RHS- Encuesta Nacional de Salud Materno Infantil 2008) MSPAS (2011)	2.5 (1.4-4.2)	7.3% (4.1-12.3)

Americas	Guyana	Guyana Women’s Health And Life Experiences Survey (2018)	3.6 (1.9-6.5)	10.7% (5.6-19.3)
Americas	Haiti	[DHS] Haïti Enquête Mortalité, Morbidité et Utilisation des Services (2018); [DHS] Haïti Enquête Mortalité, Morbidité et Utilisation des Services (2013); Demographic and Health Survey [DHS] (2007); Demographic and Health Survey [DHS] (2001)	4.1 (2.6-6.0)	12.5% (7.9-18.2)
Americas	Honduras	Encuesta Nacional De Demografía Y Salud (ENDESA/DHS) (2013); Encuesta Nacional De Demografía y Salud (ENDESA/DHS) (2006); Encuesta Nacional De Demografía y Salud (ENDESA/DHS) (2006)	2.4 (1.5-4.0)	7.0% (4.4-11.7)
Americas	Jamaica	Women's Health Survey 2016 Jamaica (Williams) (2018); Reproductive Health Survey [RHS] (2010)	2.5 (1.4-4.3)	7.3% (4.1-12.5)
Americas	Mexico	Encuesta Nacional sobre Dinámica de las Relaciones (ENDIREH) (2016); Encuesta Nacional sobre la Dinámica de las Relaciones (ENDIREH) (2013); Encuesta Nacional sobre la Dinámica de las Relaciones (ENDIREH) (2007); Encuesta Nacional sobre la Dinámica de las Relaciones (ENDIREH) (2004)	3.4 (2.1-5.3)	9.9% (6.1-15.4)
Americas	Nicaragua	Encuesta nicaragüense de demografía y salud 2011 (2014); Encuesta nicaragüense de demografía y salud 2006/2007 (2008)	2.3 (1.3-3.8)	6.7% (3.8-11.1)
Americas	Panama	Encuesta Nacional de Salud Sexual y Reproductiva (RHS) (2018); Encuesta Nacional de Salud Sexual y Reproductiva (RHS) (2011)	2.8 (1.5-4.7)	8.1% (4.4-13.6)
Americas	Paraguay	Primera encuesta sobre violencia intrafamiliar basada en genero (2014); RHS-ENDSSR-Encuesta nacional de demografía y salud sexual y reproductiva (2009); Encuesta nacional de demografía y salud sexual y reproductiva (2005); ENDSSR-Encuesta nacional de demografía y salud sexual y reproductiva (2005)	1.9 (1.0-3.3)	5.6% (2.9-9.7)
Americas	Peru	Encuesta Demográfica y de Salud Familiar ENDES [DHS] (2018); Encuesta Demográfica y de Salud Familiar ENDES [DHS] (2017); Encuesta Demográfica y de Salud Familiar ENDES [DHS] (2016); Encuesta Demográfica y de Salud Familiar ENDES [DHS] (2015); Encuesta Demográfica y de Salud Familiar ENDES [DHS] (2014); Encuesta Demográfica y de Salud Familiar ENDES [DHS] (2013); Encuesta Demográfica y de Salud Familiar ENDES [DHS] (2012); Encuesta Demográfica y de Salud Familiar ENDES [DHS] (2011); Encuesta Demográfica y de Salud Familiar ENDES [DHS] (2010); Encuesta Demográfica y de Salud Familiar ENDES [DHS] (2009); Encuesta Demográfica y de Salud Familiar ENDES [DHS] (2007); WHO Multi-Country Study (WHO MCS) (2005); Encuesta Demográfica y de Salud Familiar ENDES [DHS] (2001)	4.0 (2.9-5.4)	11.6% (8.4-15.7)
Americas	Suriname	National Women's Health Survey for Suriname (2019)	2.7 (1.4-5.0)	7.9% (4.1-14.6)
Americas	Trinidad and Tobago	National Women's Health Survey of Trinidad and Tobago (2018)	2.8 (1.4-5.1)	8.1% (4.1-14.8)
Americas	United States of America	The national intimate partner and sexual violence survey (National Centre for Injury Prevention and Control) (2017); National Longitudinal Study of Adolescent to Adult Health (2016); National Crime Victimization Survey (2016); Behavioral Risk Factor Surveillance System Survey (2014); United States National Survey of Family and Households (2012); Central Pennsylvania Women's Health Study (2012); California Women's Health Survey (2009); National Survey On Drug Use And Health (2007); Physical and Emotional Partner Abuse Reported by Men and Women in a Rural Community (Murty et al.) (2003)	2.2 (1.3-3.6)	6.4% (3.8-10.4)
Americas	Uruguay	Primera encuesta nacional de prevalencia sobre violencia basada en género y generaciones (2013)	1.6 (0.6-3.3)	4.6% (1.7-9.5)

Americas	Venezuela (Bolivarian Republic of)	Encuesta Demográfica de Venezuela 2010 (2013)	3.0 (1.5-5.7)	8.7% (4.3-16.5)
Asia	Afghanistan	Demographic and Health Survey [DHS] (2017)	11.4 (7.2-16.6)	34.3% (21.6-49.9)
Asia	Armenia	Demographic and Health Survey [DHS] (2017); Nationwide survey on domestic violence against women in Armenia (2011)	1.6 (0.7-3.1)	4.6% (2.0-8.9)
Asia	Azerbaijan	Demographic and Health Survey [DHS] (2008); Reproductive Health Survey [RHS] (2003)	1.9 (1.0-3.4)	5.5% (2.9-9.8)
Asia	Bangladesh	Report on Violence Against Women VAW Survey 2015 (2016); Report on Violence Against Women VAW Survey 2011 (2013); Demographic and Health Survey [DHS] (2009); Wife Abuse in rural Bangladesh (Dalal, K., Rahman, F. & Jansson, B.) (2009); SALAM (2006); WHO Multi-Country Study (WHO MCS) (2005)	7.9 (5.1-11.6)	23.1% (14.9-33.9)
Asia	Bhutan	Study on Situation of Violence against Women in Bhutan (2012)	2.9 (1.8-4.8)	8.6% (5.4-14.3)
Asia	Cambodia	Demographic and Health Survey [DHS] (2015); National survey on Women's Health and life experiences in Cambodia (2015); Demographic and Health Survey [DHS] (2013); Demographic and Health Survey [DHS] (2006); Demographic and Health Survey [DHS] (2001)	3.1 (1.9-4.7)	9.1% (5.6-13.9)
Asia	China	Social Staus of women in China (2014); Research on Gender Based Violence and Masculinities (Why Do Some Men Use Violence Against Women and How Can We Prevent It (UN MCS 2013) (2013); Investigation on the patterns and knowledge regarding domestic violence among married women in rural areas of China (Zhao et al.) (2006); Domestic abuse on women in China before, during, and after pregnancy (Guo, S-f., Wu, J-l., Q, C-y., Yan, R-y.) (2004)	2.8 (1.1-6.6)	8.1% (3.2-19.0)
Asia	Georgia	National Study of Violence Against Women in Georgia [UN Women/GEOSTAT] (2018)	1.0 (0.4-2.2)	2.9% (1.2-6.4)
Asia	India	National Family Health Survey/Demographic And Health Survey (NFHS/DHS) (2017); Physical wife abuse in an urban slum of Pune, Maharastra (Ruikar, MM., Pratinidhi, AK) (2008); National Family Health Survey/Demographic And Health Survey (NFHS/DHS) (2007); National Family Health Survey/Demographic And Health Survey (NFHS/DHS) (2004); World Studies of Abuse in the Family Environment (WorldSAFE) (2004)	6.2 (3.8-9.6)	18.3% (11.2-28.3)
Asia	Indonesia	Study on Women's and Men's Health and Life Experiences in Papua, Indonesia (UNDP) (2016)	3.2 (1.3-7.1)	9.4% (3.8-20.8)
Asia	Iran (Islamic Republic of)	Domestic Abuse in Behshahr, Iran (Rahmatian, A.A. & Hosseini, S.A.A.) (2015); Risk Factors of Domestic Violence in Iran (Rasoulin, M., et al.) (2014); Domestic violence: a cross-sectional study in an Iranian city (Ghazizadeh, A.) (2005); Wife abuse in Esfahan, Islamic Republic of Iran, 2002 (Mousavi, SM. & A. Eshagian) (2005)	5.9 (2.5-12.4)	17.0% (7.2-35.8)
Asia	Israel	Is elder abuse and neglect a social phenomenon? Data from the First National Prevalence Survey in Israel (Lowenstein, A., Eisikovits, Z., Band-Winterstein, T., Enosh, G.) (2009); The First Israeli National Survey on Domestic Violence (Eisikovits, Z., Winstok, Z., Fishman, G.) (2004)	2.0 (1.0-3.8)	5.7% (2.9-10.9)
Asia	Japan	WHO Multi-Country Study (WHO MCS) (2005); HASEGAWA (2005)	1.5 (0.5-3.9)	4.3% (1.4-11.2)
Asia	Jordan	Jordan Population and Family Health Survey/ Demographic and Health Survey (DHS) (2019); Jordan Population and Family Health Survey/ Demographic and Health Survey (DHS) (2013); Jordan Poulation and	4.5 (3.0-7.0)	13.0% (8.7-20.3)

		Family Health Survey/ Demographic and Health Survey (DHS) (2008)		
Asia	Kazakhstan	Ministry of National Economy of the Republic of Kazakhstan (2017)	2.0 (1.0-4.0)	5.8% (2.9-11.7)
Asia	Kyrgyzstan	Demographic and Health Survey [DHS] (2013)	4.3 (2.3-7.8)	12.5% (6.7-22.7)
Asia	Lao People's Democratic Republic	Lao National Survey on Women's Health and Life Experiences 2014 (2015)	2.8 (1.4-5.2)	8.3% (4.2-15.4)
Asia	Maldives	Demographic and Health Survey [DHS] (2018); Maldives Study on Women's Health and Life Experiences (2011)	2.2 (1.3-3.9)	6.3% (3.7-11.2)
Asia	Mongolia	National Study on Gender-based Violence in Mongolia (2018)	3.8 (2.1-6.7)	11.0% (6.1-19.4)
Asia	Myanmar	Demographic and Health Survey [DHS] (2017); Prevalence, antecedent causes and consequences of domestic violence in Myanmar (Kyu, N. & Kana, A.) (2005)	3.9 (2.1-6.8)	11.5% (6.2-20.1)
Asia	Nepal	Demographic and Health Survey [DHS] (2017); Demographic and Health Survey [DHS] (2012)	3.9 (2.3-6.4)	11.5% (6.8-18.8)
Asia	Pakistan	Demographic and Health Survey [DHS] (2019); Demographic and Health Survey [DHS] (2013)	5.5 (3.4-8.6)	16.1% (10.0-25.2)
Asia	Philippines	Demographic and Health Survey [DHS] (2018); Demographic and Health Survey [DHS] (2014); Demographic and Health Survey [DHS] (2009); International Violence Against Women Survey (IVAWS) (2008); Cebu Longitudinal Health And Nutrition Survey (2008); World Studies of Abuse in the Family Environment (WorldSAFE) (2004)	2.2 (1.3-3.4)	6.4% (3.8-9.9)
Asia	Republic of Korea	The Domestic Violence Survey in 2016 (2016); Prevalence and Trends in Domestic Violence in South Korea: Findings From National Surveys (Kim, Oh and Nam) (2015); Prevalence and Trends in Domestic Violence in South Korea: Findings From National Surveys (Kim, Oh and Nam) (2015)	2.9 (1.5-5.0)	8.3% (4.3-14.4)
Asia	Singapore	International Violence Against Women Survey (IVAWS) (2013)	0.9 (0.4-2.2)	2.6% (1.1-6.3)
Asia	Sri Lanka	Why Do Some Men Use Violence Against Women And How Can We Prevent It (UN MCS 2013) (2013)	1.5 (0.6-3.5)	4.3% (1.7-10.1)
Asia	State of Palestine	Palestinian National Survey On Violence Against Women (2012)	6.4 (3.5-11.1)	18.6% (10.1-32.2)
Asia	Tajikistan	Demographic and Health Survey [DHS] (2018); Demographic and Health Survey [DHS] (2013)	4.5 (2.7-7.2)	13.0% (7.8-20.9)
Asia	Thailand	Intimate partner violence among women in slum communities in Bangkok, Thailand (Aekplakorn, W & Kongsakon, R) (2007); WHO Multi-Country Study (WHO MCS) (2005)	3.4 (1.5-7.5)	9.9% (4.4-21.8)
Asia	Timor-Leste	Demographic and Health Survey [DHS] (2018); Findings from the Nabilan Baseline Study Understanding Violence against Women and Children in Timor-Leste: (2016); Demographic and Health Survey [DHS] (2010); A determination of the prevalence of gender-based violence among conflict-affected populations in East Timor (2004)	9.4 (6.3-13.5)	27.5% (18.4-39.4)
Asia	Turkey	Ethnic Background And Alcohol Use Of The Spouse Emerge As Major Risk Factors For Domestic Violence: An Observational Study From Turkey (Guclu, YA. & Can H.) (2018); Research on Domestic Violence against Women in Turkey (2015); What Puts Women at Risk of Violence From Their Husbands? Findings From a Large, Nationally Representative Survey in Turkey (2012); National Research on Domestic Violence against Women in Turkey (2009)	4.3 (2.7-6.9)	12.5% (7.8-20.0)

Asia	Viet Nam	Primary Health Improvement Initiative (2012); National Study On Domestic Violence Against Women In Viet Nam (2010); Filabavi Surveillance (2008)	3.5 (1.9-6.2)	10.1% (5.5-17.9)
Europe	Albania	National Population Survey: Violence against Women and Girls in Albania (INSTAT-SVIERGE-UNDP-UNW) (2019); Well-being and Safety of Women (OSCE) (2019); Demographic and Health Survey [DHS] (2018); Domestic Violence in Albania National population -based Survey (2013); Reproductive Health Survey [RHS] (2005)	2.1 (1.5-3.1)	6.1% (4.3-8.9)
Europe	Austria	Violence against women: an EU-wide survey (FRA) (2014)	1.3 (0.6-2.7)	3.7% (1.7-7.8)
Europe	Belarus	Prevalence of violence for women in the Republic of Belarus: Family Safety Study in the Republic of Belarus (2019)	2.2 (1.0-4.1)	6.4% (2.9-11.9)
Europe	Belgium	Etude de prévalence sur les violences faites aux femmes en Région de Bruxelles-capitale (2017); Violence against women: an EU-wide survey (FRA) (2014); Emotional, physical and sexual abuse - the experiences of women and men (2010)	1.7 (1-3.2)	4.9% (2.9-9.2)
Europe	Bosnia and Herzegovina	Well-being and Safety of Women (OSCE) (2019); Prevalence and characteristics of violence against women in BIH (2013)	1.2 (0.7-2.2)	3.4% (2.0-6.3)
Europe	Bulgaria	Violence against women: an EU-wide survey (FRA) (2014)	2.1 (1.0-4.2)	6.1% (2.9-12.1)
Europe	Croatia	Violence against women: an EU-wide survey (FRA) (2014)	1.4 (0.6-3.0)	4.0% (1.7-8.6)
Europe	Cyprus	Violence against women: an EU-wide survey (FRA) (2014); Extent, Frequency, Nature and Consequences of Domestic Violence Against Women in Cyprus (2012)	1.2 (0.4-2.4)	3.4% (1.1-6.9)
Europe	Czechia	Abused, Battered, or Stalked: Violence in Intimate Partner Relations Gendered (Buriánek, Pikálková, Podaná, and Kolínská: IVAWS follow-up) (2016); Violence against women: an EU-wide survey (FRA) (2014); International Violence Against Women Survey (IVAWS) (2008)	1.5 (0.8-2.4)	4.3% (2.3-6.9)
Europe	Denmark	Violence and Sexual Abuse, National Institute of Public Health, Denmark (2018); Violence against women: an EU-wide survey (FRA) (2014); International Violence Against Women Survey (IVAWS) (2008)	1.2 (0.7-2.0)	3.4% (2.0-5.7)
Europe	Estonia	Violence against women: an EU-wide survey (FRA) (2014)	1.5 (0.6-3.2)	4.3% (1.7-9.2)
Europe	Finland	Institute of Criminology and Legal Policy, University of Helsinki (2019); Institute of Criminology and Legal Policy, University of Helsinki (2018); Institute of Criminology and Legal Policy, University of Helsinki (2017); Institute of Criminology and Legal Policy, University of Helsinki (2016); Institute of Criminology and Legal Policy, University of Helsinki (2015); Institute of Criminology and Legal Policy, University of Helsinki (2014); Violence against women: an EU-wide survey (FRA) (2014); Violence Against Women in Finland (Heiskanen, M. & Piipsa, M.) (2008)	2.9 (2.0-4.0)	8.3% (5.8-11.5)
Europe	France	Violence against women: an EU-wide survey (FRA) (2014); Les violence contre les femmes (Maryse Jaspard) (2001)	1.8 (0.9-3.7)	5.2% (2.6-10.6)
Europe	Greece	Violence against women: an EU-wide survey (FRA) (2014)	1.9 (0.9-3.7)	5.5% (2.6-10.6)
Europe	Hungary	Violence against women: an EU-wide survey (FRA) (2014)	2.0 (1.0-4.0)	5.8% (2.9-11.5)
Europe	Iceland	European Institute for Crime Prevention and Control (HEUNI) (2018); European Institute for Crime Prevention and Control (HEUNI) (2017); European Institute for Crime Prevention and Control (HEUNI) (2016); European Institute for Crime Prevention and Control (HEUNI) (2015); Ingólfur Gíslason,Ministry for Welfare (2009)	0.9 (0.6-1.6)	2.6% (1.7-4.6)

Europe	Ireland	Violence against women: an EU-wide survey (FRA) (2014); Domestic Abuse of Women and Men in Ireland: Report on the National Study of Domestic Abuse (The National Crime Council in association with the Economic and Social Research Institute (ESRI)/Watson)) (2005)	1.2 (0.6-2.3)	3.5% (1.7-6.6)
Europe	Italy	Violence and abuses against women inside and outside family (ISTAT) (2015); Violence against women: an EU-wide survey (FRA) (2014); Violence and abuses against women inside and outside family (ISTAT) (2006)	1.3 (0.8-2.3)	3.7% (2.3-6.6)
Europe	Latvia	Violence against women: an EU-wide survey (FRA) (2014)	2.2 (1.0-4.3)	6.4% (2.9-12.4)
Europe	Lithuania	Violence against women: an EU-wide survey (FRA) (2014); Schrottle (2003)	1.8 (0.8-3.7)	5.2% (2.3-10.7)
Europe	Luxembourg	Violence against women: an EU-wide survey (FRA) (2014)	1.3 (0.5-2.7)	3.7% (1.4-7.8)
Europe	Malta	Violence against women: an EU-wide survey (FRA) (2014); The prevalence of domestic violence against women in Malta (2011)	1.4 (0.7-3.0)	4.0% (2.0-8.6)
Europe	Montenegro	Well-being and Safety of Women (OSCE) (2019); Study on Family Violence and Violence against Women in Montenegro (2012)	1.5 (0.7-2.9)	4.3% (2.0-8.4)
Europe	Netherlands	Violence against women: an EU-wide survey (FRA) (2014)	1.8 (0.9-3.6)	5.2% (2.6-10.3)
Europe	North Macedonia	Well-being and Safety of Women (OSCE) (2019)	1.5 (0.7-3.0)	4.3% (2.0-8.6)
Europe	Norway	Partner violence and health: results from the first national study on violence against women in Norway (Astrid Irene Nerøien and Berit Schei) (2008)	1.5 (0.7-3.1)	4.3% (2.0-8.9)
Europe	Poland	Violence against women: an EU-wide survey (FRA) (2014); International Violence Against Women Survey (IVAWS) (2008)	1.0 (0.6-2.0)	2.9% (1.7-5.8)
Europe	Portugal	Violence against women: an EU-wide survey (FRA) (2014)	1.6 (0.8-3.2)	4.6% (2.3-9.2)
Europe	Republic of Moldova	Well-being and Safety of Women (OSCE) (2019); Violence against women in the family in Moldova (2011); Demographic and Health Survey [DHS] (2006)	3.0 (1.7-5.2)	8.7% (4.9-15.0)
Europe	Romania	Violence against women: an EU-wide survey (FRA) (2014); Reproductive Health Survey [RHS] (2005)	2.3 (1.3-4.0)	6.6% (3.7-11.5)
Europe	Serbia	Well-being and Safety of Women (OSCE) (2019); WHO Multi-Country Study (WHO MCS) (2005)	1.3 (0.7-2.7)	3.7% (2.0-7.8)
Europe	Slovakia	Violence against women: an EU-wide survey (FRA) (2014)	2.0 (1.0-4.0)	5.8% (2.9-11.5)
Europe	Slovenia	Violence against women: an EU-wide survey (FRA) (2014); Survey on Violence Against Women in the private sphere and partnership relations conducted in 2010 (2010)	1.2 (0.6-2.3)	3.4% (1.7-6.6)
Europe	Spain	Macroencuesta De Violencia Contra La Mujer 2015 (2015); Violence against women: an EU-wide survey (FRA) (2014); Análisis Sobre La Macroencuesta De Violencia De Género 2011 (2012)	1.1 (0.7-1.8)	3.2% (2.0-5.2)
Europe	Sweden	Violence against women: an EU-wide survey (FRA) (2014); Self-reported exposure to intimate partner violence among women and men in Sweden: results from a population-based survey (Nybergh, L., Taft, C., Enander, V. & Krantz, G.) (2013); Captured Queen — Men's violence in "equal" Sweden-a prevalence study (Lundgren, E., Heimer, G., Westersrand, J., Kalliokoski, A-M.) (2001)	2.3 (1.2-4.1)	6.6% (3.4-11.8)
Europe	Switzerland	International Violence Against Women Survey (IVAWS) (2008)	0.7 (0.3-1.4)	2.0% (0.9-4.0)
Europe	Ukraine	Well-being and Safety of Women (OSCE) (2019); The prevalence of domestic violence against women and girls (GfK Ukraine) (2014); Demographic and Health Survey [DHS] (2008); World Mental Health (WMH) Survey (2008)	2.9 (1.7-4.6)	8.4% (4.9-13.4)

Europe	United Kingdom	Violence against women: an EU-wide survey (FRA) (2014); Adult Psychiatric Morbidity Survey (2014)	1.5 (0.8-3.1)	4.3% (2.3-8.9)
Oceania	Australia	Personal Safety Survey (2017); Personal Safety Survey (2013); International Violence Against Women Survey (IVAWS) (2008); Personal Safety Survey (2006); Melbourne Women's Midlife Health Project (2006); International Violence Against Women Survey (IVAWS) (Mouzos J. & Makai, T.) (2004)	1.0 (0.6-1.7)	2.9% (1.7-4.9)
Oceania	Fiji	National Research on Women's Health and Life Experiences in Fiji (Fiji Women's Crisis Centre) (2013)	8.2 (4.6-13.5)	24.4% (13.7-40.2)
Oceania	Kiribati	Kiribati Family Health and Support Study (2010)	8.6 (4.6-14.8)	25.3% (13.5-43.5)
Oceania	Micronesia (Fed. States of)	Federated States of Micronesia Family Health and Safety Study (2014)	7.4 (4.0-12.6)	21.8% (11.8-37.0)
Oceania	New Zealand	WHO Multi-Country Study (WHO MCS) (2005)	1.6 (0.5-4.0)	4.6% (1.4-11.5)
Oceania	Papua New Guinea	Demographic and Health Survey [DHS] (2019); Why Do Some Men Use Violence Against Women And How Can We Prevent It (UN MCS 2013) (2013)	10.3 (6.3-15.4)	30.7% (18.8-45.9)
Oceania	Samoa	WHO Multi-Country Study (WHO MCS) (2005)	6.5 (3.4-11.6)	18.8% (9.8-33.5)
Oceania	Solomon Islands	Solomon Islands Family Health and Safety Study (2009)	9.6 (5.2-16.0)	28.2% (15.3-46.9)
Oceania	Tonga	National Study On Domestic Violence Against Women In Tonga (2012)	6.1 (3.1-11.2)	17.8% (9.0-32.7)
Oceania	Vanuatu	Vanuatu National Survey on Women’s Lives and Family Relationships (2011)	10.1 (5.4-16.7)	29.3% (15.7-48.5)

Note:
*Countries with less than 100 thousand population were excluded from the analysis to avoid volatility of the results: Cook Islands, Marshal Islands, Nauru, Palau, and Tuvalu

Source: Authors’ elaboration.

4. Appendix references:

1. United Nations – Department of Economic and Social Affairs. Annual Population by Age – Female [Internet] World Population Prospects. 2019 [cited 16 June 2020] Available from: https://population.un.org/wpp/Download/Standard/Interpolated/
2. United Nations - Department of Economic and Social Affairs. Estimates and projections of women of reproductive age who are married or in a Union: 2020 Revision. 2020 [cited 26 July 2020] Available from: https://www.un.org/en/development/desa/population/theme/marriage-unions/marriage_estimates.asp
3. WHO – World Health Organization. Global Database on the Prevalence of Violence Against Women. National estimates 2000 – 2018. [Internet] WHO-World Health Organization. 2021 [cited 05 July 2021] Available from: https://srhr.org/vaw-data
